# An unusual case of primary choriocarcinoma of the lung

**DOI:** 10.1186/1471-2482-13-S2-S33

**Published:** 2013-10-08

**Authors:** Vincenzo Di Crescenzo, Paolo Laperuta, Filomena Napolitano, Chiara Carlomagno, Alfredo Garzi, Mario Vitale

**Affiliations:** 1 Department of Medicine and Surgery, University of Salerno, Italy; 2 Department of Clinical Medicine and Surgery (2), University of Naples "Federico II", Italy

**Keywords:** Choriocarcinoma, Lung

## Abstract

Choriocarcinoma is a germ cell tumor containing syncytiotrophoblastic cells and secreting human Beta-HCG. Primary choriocarcinoma of the lung is extremely uncommon. The prognosis of this tumor is extremely poor, despite surgical and chemotherapeutic treatment. We report a surgically treated case of choriocarcinoma in a 37-year-old woman who came to our attention because of a isolated lung lesion. The tumor was successfully resected. Chemotherapy was started 2 months after thoracic surgery and consisted of bleomycin, etoposide, and cisplatin. At 1-year follow-up the patient is alive in good condition. The hCG level is actually normal.

## Background

Choriocarcinoma is a germ cell tumor containing syncytiotrophoblastic cells and secreting human Beta-HCG. Primary choriocarcinoma (PCC) of the lung is extremely uncommon. The prognosis of this tumor is extremely poor, despite surgical and chemotherapeutic treatment [1-3]. We report a surgically treated case of choriocarcinoma in a 37-year-old woman who came to our attention because of a isolated lung lesion.

## Clinical presentation

**Figure 1:**
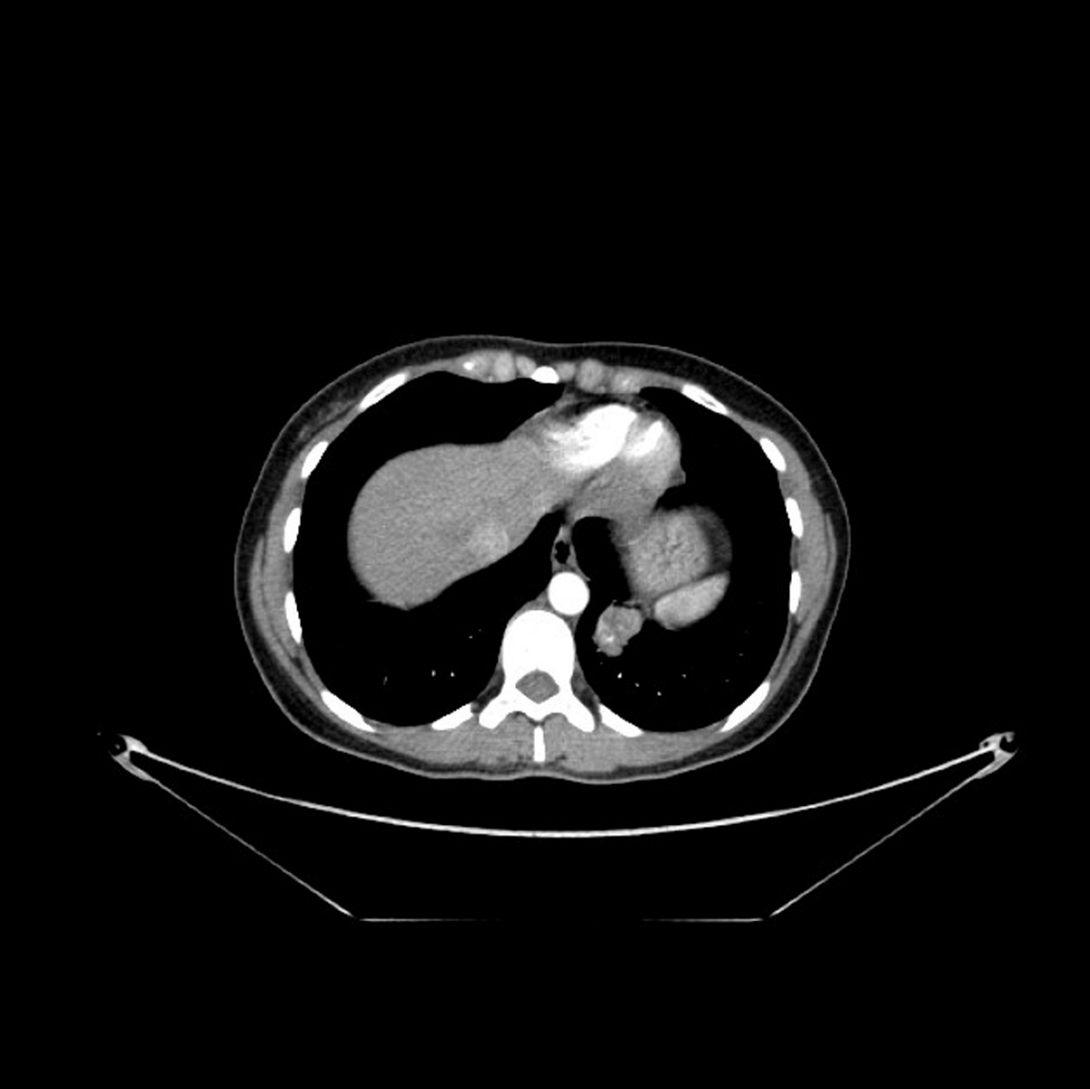
**Chest computed tomography (CT) scan demonstrated an 4 cm pulmonary mass in the left lower lobe, without enlargement of intrapulmonary or mediastinal lymph nodes**.

**Figure 2:**
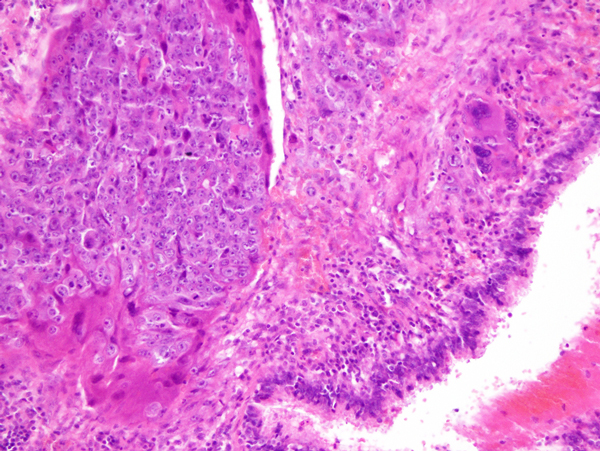
**Histological results showed that the tumor was characteristic of choriocarcinoma, with large multinucleated cells representing syncytiotrophoblast admixed with medium-sized cells, often with clear cytoplasm, similar to cytotrophoblasts or intermediate trophoblasts (Hematoxilin Eosin 430X)**.

A 37 year-old woman complained of hemoptosis and dyspnea was admitted to our hospital. Routine laboratory tests showed no abnormalities. Elevated serum tumor markers were within normal values except of human chorionic gonadotropin (hCG) that was elevated (2686.54 mIU/ml). A pregnancy was suspected, but the urinary pregnancy test was found negative and ultra sound evaluation did not show any direct or indirect sign of pregnancy. Chest computed tomography (CT) scan showed an 4 cm pulmonary mass in the left lower lobe, without enlargement of intrapulmonary or mediastinal lymph nodes (Figure 1). Screening for additional masses including CT of the abdomen and brain as well as bone scintigraphy showed no abnormalities. At ^18^FDG-PET the lung mass was FDG-avid (SUV: 7.5). Bronchoscopy showed no endoluminal lesion and the pathological results from bronchoalveolar lavage showed the presence of inflammatory cells in absence of malignant cells [4,5]. A FNAB-CT guided was done; the results showed the presence of atypical cell suggestive for malignancy but inconclusive for definitive diagnosis. Cardio-respiratory functions did not contraindicated surgery. Thus, a lower left lobectomy with a conventional mediastinal node dissection was attended. A PCA was performed for controlling post-operative pain [6]. The histological results are reported in Figure 2. Microscopically, the tumor was characteristic of choriocarcinoma, with large multinucleated cells representing syncytiotrophoblast admixed with medium-sized cells, often with clear cytoplasm, similar to cytotrophoblasts or intermediate trophoblasts (Figure n.2). Immunohistochemically, the tumor cells were reactive for hCG and cytokeratin AE1/AE3, but not for CEA. The tumor therefore was diagnosed pathologically as choriocarcinoma. Serum hCG concentrations fell to 14.4 mIU/ml during the second postoperative week. In consideration of the various findings, the patient was diagnosed with PCC of the lung.

After operation an intensive systemic workup carried out in search of an occult choriocarcinoma was negative for tumor; including hysteorosalpingo-oophorectomy, the digestive organs, the reproductive tract and the surrounding tissues were all intact.

Chemotherapy was started 2 months after thoracic surgery and consisted of bleomycin, etoposide, and cisplatin. At 1-year follow-up the patient is alive in good condition. The hCG level is actually normal.

## Discussion

Choriocarcinoma is a malignant proliferation of the Langerhans cell and of syncytial cells of trophoblastic origin that is normally situated in the female genital tract after a gestational event such as molar pregnancy, term pregnancy, abortion, or ectopic pregnancy [1,2].

Primary extragenital choriocarcinoma most often arises in the retroperitoneum, in the mediastinum, or intracranially. Although metastasis to the lung is not infrequent, PCC originating in the lung is extremely rare. The rarity of the occurrence of PPC and the small size of tumour samples make it challenging to diagnose either by cytology or bronchoscopic biopsy alone. The prognosis of extragonadal choriocarcinoma is usually poor, with various symptoms seriously affecting quality of life. Actually, resection followed by adjuvant chemotherapy appears to represent the best treatment for PCC of the lung [3]

In the present we firstly suspected a pregnancy in the light of high hCG value. Because all laboratory and diagnostic tests resulted to be negative, a chest CT scan was attended that showed the presence of lung mass within left lower lobe. Bronchoscopy showed no endobronchial lesion n and the results of bronchoalveolar lavage showed inflammatory cells without malignant characteristics [6-11]. The results of FNAB was suggestive for a lung cancer. Thus a standard lower left lobectomy was attended. Surprisingly, the histological results diagnosed the tumor to be a choriocarcinoma [1,2]. In the present case, PCC of the lung was diagnosed after operation on the basis of the following observations: hCG fell dramatically after lobectomy: the lesion was limited to the lung, the patient's clinical course after surgery was uneventful, the ovaries, uterus and uterine tubes were found to be free of lesions on surgical removal, and no lesions were found in the digestive system.

Several explanations might be offered for this occurrence of PCC in the lung: origin from retained primordial germ cells that migrated abnormally during embryonic development; metastasis from a primary gonadal tumor that regressed spontaneously; or origin from trophoblastic emboli related to molar pregnancy after a long period of latency. Other reports support a hypothesis of dedifferentiation or metaplasia of nongonadal tissue such as primary lung cancer to trophoblast [1-3].

In closure, a positive hCG test result in patients with hemoptysis and progressive dyspnea could be diagnostic for a pulmonary choriocarcinoma and may be helpful for a early diagnosis. The diagnostic criteria would include lack of a previous gynecologic malignancy, solitary or predominant lung lesion with the exclusion of a gonadal primary site, raised serum hCG titers that become normal after surgery or chemotherapy, and pathologic confirmation of the disease

## Competing interests

The authors declare that they have no competing interests.

## Declarations

Funding for publication of the article has come from the: POMPEANGAS Via S. Sebastiano n. 16, ANGRI (SA) Italia. P.IVA 02600816055

This article has been published as part of *BMC Surgery *Volume 13 Supplement 2, 2013: Proceedings from the 26th National Congress of the Italian Society of Geriatric Surgery. The full contents of the supplement are available online at http://www.biomedcentral.com/bmcsurg/supplements/13/S2

## References

[B1] Serno J Zeppernick F Jäkel J Schrading S Maass N Meinhold-Heerlein I Bauerschlag DO Primary pulmonary choriocarcinoma: case report and review of the literature Gynecol Obstet Invest 2012 74 171 176 22738859 10.1159/000336784

[B2] Berthod G Bouzourene H Pachinger C Peters S Solitary choriocarcinoma in the lung J Thorac Oncol 2010 5 574 575 20357625 10.1097/JTO.0b013e3181cbf372

[B3] Berthod G Bouzourene H Pachinger C Peters S Solitary choriocarcinoma in the lung J Thorac Oncol 2010 5 574 575 20357625 10.1097/JTO.0b013e3181cbf372

[B4] Fiorelli A Morgillo F Fasano M Vicidomini G Di Crescenzo VG Di Domenico M Accardo M Santini M The value of matrix metalloproteinase-9 and vascular endothelial growth factor receptor 1 pathway in diagnosing indeterminate pleural effusion Interact Cardiovasc Thorac Surg 2013 16 263 269 23190621 10.1093/icvts/ivs466 PMC3568795

[B5] Santini M Fiorelli A Vicidomini G Laperuta P Di Crescenzo VG Iatrogenic air leak successfully treated by bronchoscopic placement of unidirectional endobronchial valves Ann Thorac Surg 2010 89 2007 2010 20494069 10.1016/j.athoracsur.2009.10.015

[B6] Santini M Fiorello A Di Crescenzo VG Vicidomini G Busiello L Laperuta P Use of unidirectional endobronchial valves for the treatment of giant emphysematous bulla J Thorac Cardiovasc Surg 2010 139 224 226 19660316 10.1016/j.jtcvs.2008.05.069

[B7] Santini M Fiorelli A Vicidomini G Di Crescenzo VG Messina G Laperuta P Endobronchial treatment of giant emphysematous bullae with one-way valves: a new approach for surgically unfit patients Eur J Cardiothorac Surg 2011 40 1425 1431 21764325 10.1016/j.ejcts.2011.03.046

[B8] Baldi A Mottolese M Vincenzi B Campioni M Mellone P Di Marino M di Crescenzo VG Visca P Menegozzo S Spugnini EP Citro G Ceribelli A Mirri A Chien J Shridhar V Ehrmann M Santini M Facciolo F The serine protease HtrA1 is a novel prognostic factor for human mesothelioma Pharmacogenomics 2008 9 1069 1077 18681782 10.2217/14622416.9.8.1069

[B9] Santini M Fiorello A Vicidomini G Di Crescenzo VG Laperuta P Role of diffusing capacity in predicting complications after lung resection for cancer Thorac Cardiovasc Surg 2007 55 6 391 4 17721850 10.1055/s-2007-965326

[B10] Napolitano V Pezzullo AM Zeppa P Schettino P D'Armiento M Palazzo A Della Pietra C Napolitano S Conzo G Foregut duplication of the stomach diagnosed by endoscopic ultrasound guided fine-needle aspiration cytology: case report and literature review World J Surg Oncol 2013 11 33 Feb 2 23374143 10.1186/1477-7819-11-33 PMC3599514

[B11] Zeppa P Varone V Cozzolino I Salvatore D Vetrani A Palombini L Fine needle cytology and flow cytometry of ectopic cervical thymoma: a case report Acta Cytol 2010 54 998 1002 21053586

